# Room-temperature solution-phase epitaxial nucleation of PbS quantum dots on rutile TiO_2_ (100)†

**DOI:** 10.1039/c9na00601j

**Published:** 2019-12-03

**Authors:** Stefan Kraus, Mischa Bonn, Enrique Cánovas

**Affiliations:** Max Planck Institute for Polymer Research Ackermannweg 10 55128 Mainz Germany; Instituto Madrileño de Estudios Avanzados en Nanociencia (IMDEA Nanociencia) Faraday 9 28049 Madrid Spain enrique.canovas@imdea.org

## Abstract

Owing to its simplicity and versatility, the successive ionic layer adsorption and reaction (SILAR) method is increasingly being employed to develop low-cost hetero-nanostructured sensitized oxide systems for solar energy conversion, such as solar cells and solar fuels schemes. Understanding the nature of the SILAR quantum dot (QD) nucleation and growth on an insulating oxide is then critical as it will determine the QD density and spatial distribution, as well as the optoelectronic properties of the QD/oxide interfaces (*e.g.* QD bandgap onset). Here, we demonstrate epitaxial nucleation of lead sulfide (PbS) QDs onto a planar rutile titanium dioxide (100) surface employing the SILAR method. The QDs nucleated by SILAR are crystalline structures characterized by a truncated pyramidal shape, with nucleation occurring preferentially along the rutile (010) and (001) crystal orientations. The PbS QD size distribution is constrained by lattice mismatch causing strain in the lead sulfide. These results highlight the potential of SILAR for the facile growth of high-quality epitaxial nanostructures in liquid phase, under ambient conditions and at room temperature.

## Abstract 2

We demonstrate epitaxial nucleation of QDs onto titania by the successive ionic layer adsorption and reaction (SILAR) method at room temperature.

## Graphical abstract

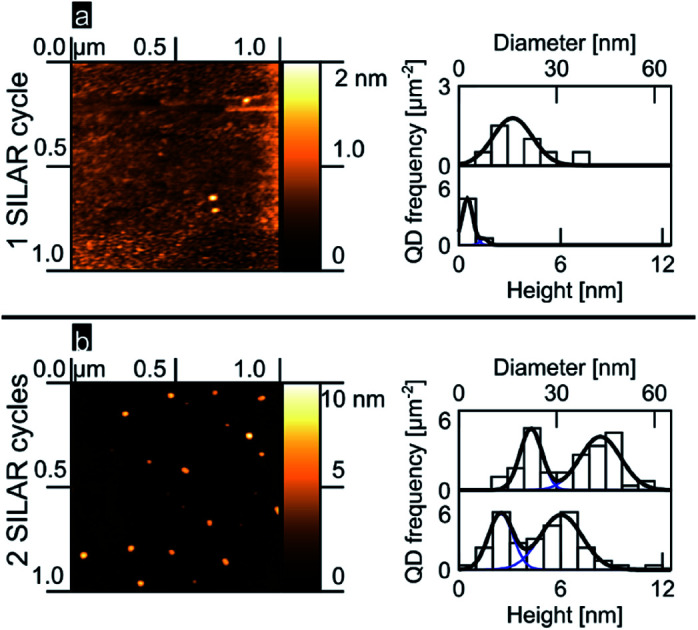

## Introduction

The epitaxial growth of nanocrystals onto semiconductor surfaces represents an appealing path towards the integration of quantum dots (QDs) in optoelectronic devices. Indeed, QDs grown by molecular beam epitaxy (MBE) and chemical vapor deposition (CVD) have been successfully exploited in all-solid-state QD based lasers and solar cells.^1–3^ Epitaxial QD growth onto semiconducting substrates has been shown to fundamentally depend on the amount of deposited material and the lattice mismatch between QD and substrate.^4,5^ The lattice mismatch ultimately determines whether epitaxial QD nucleation is feasible: (i) if there is little-to-no lattice mismatch between deposited material and substrate, the epitaxial growth provides flat crystalline thin films, the so-called Frank-van der Merwe (FM) growth, characteristic for homo-junctions, for example; (ii) as the lattice mismatch between deposited material and substrate increases, QD nucleation occurs in the so-called Volmer–Weber (VW) and Stranski–Krastanov (SK) growth regimes, which can be experimentally distinguished by the absence or presence, respectively, of a wetting layer underneath the nucleated QDs. For these regimes, growth, and ripening of quantum dots occurs from a characteristic threshold amount of deposited material.^6^ QD ripening is manifested during epitaxial growth as a transition from monomodal to bimodal QD size distributions.^6^ These characteristics of epitaxial growth have been experimentally observed in many systems obtained through MBE and CVD.^7–20^

Although the control of QD nucleation has reached exquisite levels for gas-phase deposition techniques in ultrahigh vacuum, where the effects of strain, composition and shape transitions have been successfully characterized and modelled,^2,3,7–10,21^ a drawback of these approaches is the high cost associated with these methods. Alternatively, low-cost solution-processed approaches have shown their potential for growing nanocrystals onto semiconducting and insulating surfaces at room temperature.^22,23^ For example, chemical bath deposition (CBD)^24^ and successive ionic layer adsorption and reaction (SILAR) have become popular methods for developing QD-sensitized mesoporous oxide architectures exploited in solar energy conversion schemes (solar cells and solar fuels).^25–29^ Among these approaches, the SILAR method seems particularly appealing due to the enhanced control on the deposition, enabled by the sequential deposition of anionic and cationic precursor species. Indeed, SILAR has allowed for the deposition of binary and ternary bulk and polycrystalline semiconducting thin films.^30–33^ Moreover, SILAR has been successfully employed for capping colloidal quantum dots with an epitaxially-grown lattice-matched shell of tunable thickness;^34–36^ SILAR has further allowed fine-tuning of QD surface stoichiometry (*e.g.* exploiting atomic passivation schemes)^37^ and doping of QDs.^38,39^ For QD-sensitized mesoporous oxide systems, controlling the number of SILAR cycles (deposition steps) and solution chemistry (concentration of precursors, complexing agents, *etc.*) has allowed tuning the QD optical bandgap and even the number of nanocrystals per substrate unit area.^25,27,40–42^ Although several authors have reported SILAR QD-based nucleation signatures consistent with epitaxial growth (for QDs grown on TiO_2_, ZnO and SnO_2_),^26,37,40,43^ the nature of the QD SILAR nucleation onto an insulating oxide, whether epitaxial or not, has not yet been addressed and has remained an open question.^23^ Understanding this feature is critical for device applications due to the fact that the strain accumulated in epitaxially grown QDs can be used as an extra knob for fine-tuning their optoelectronic properties (*e.g.* bandgap onset).^20,44,45^ Note that in principle different crystal facets for a given metal oxide might enable different kinetics of nucleation, which will be strongly dependent on the set of materials employed and specific growth recipe, and these will co-determine the opto-electronic properties for the sensitized system.^46–48^

Here we demonstrate the epitaxial nature of PbS QDs nucleated on planar TiO_2_ rutile (100) by SILAR; a technologically relevant sensitized system.^49–51^ A distinct and defining characteristic of epitaxial growth is resolved, the observation of crystalline PbS QDs (shaped like truncated pyramids) which are preferentially oriented on the oxide surface. The results presented here reveal the potential of SILAR for growing epitaxial nanostructures at low cost (in liquid phase, under ambient conditions, and at room temperature) and illustrate the need of considering donor–acceptor interfacial strain effects when modeling and characterizing the optoelectronic properties of QD sensitized oxides grown by *in situ* nucleation methods.

## Results and discussion

In this work, we focus our analysis on the crystallographic QD-substrate lattice mismatch (the strain energy component). While this approach is based purely on geometry, it is capable of revealing the epitaxial nature of the system based on the coherence of nucleated islands,^4,5,7,9,52,53^ For a full description of the nucleation process (not intended in this work), surface and interfacial energetic contributions to the nucleation should be considered.^54–57^

The lattice mismatch *ε* of material *A* grown on substrate *B* can be easily calculated from the lattice constants d*A* and d*B* as *ε* = (d*A* − d*B*)/d*B*.^4,5^ For the growth of PbS onto TiO_2_ rutile (100) we infer lattice mismatches of 29% and 101% for the rutile *b*- and *a*-axis respectively.

PbS QDs were nucleated onto (100) TiO_2_ single crystal substrates, 5 × 5 mm^2^ area, by following a two SILAR cycle recipe. One SILAR cycle is defined here as the successive immersion of the substrate in lead nitrate (PbNO_3_) methanol solution for 20 s, 30 s immersion rinsing in methanol, 20 s immersion in sodium sulfide (Na_2_S) methanol solution, and 30 s immersion rinsing in methanol. Precursor concentrations of 20 mmol L^−1^ were selected as representative of those typically used for sensitizing large area mesoporous oxides.^26,28,30,31,58–60^ The SILAR procedure was carried out in a nitrogen-purged glove box. Samples were characterized with tapping mode atomic force microscopy (AFM) in air, with a lateral resolution defined by the 7 nm AFM tip radius.^61^ Further details of the preparation method are given in the Experimental section.

**Fig. 1:**
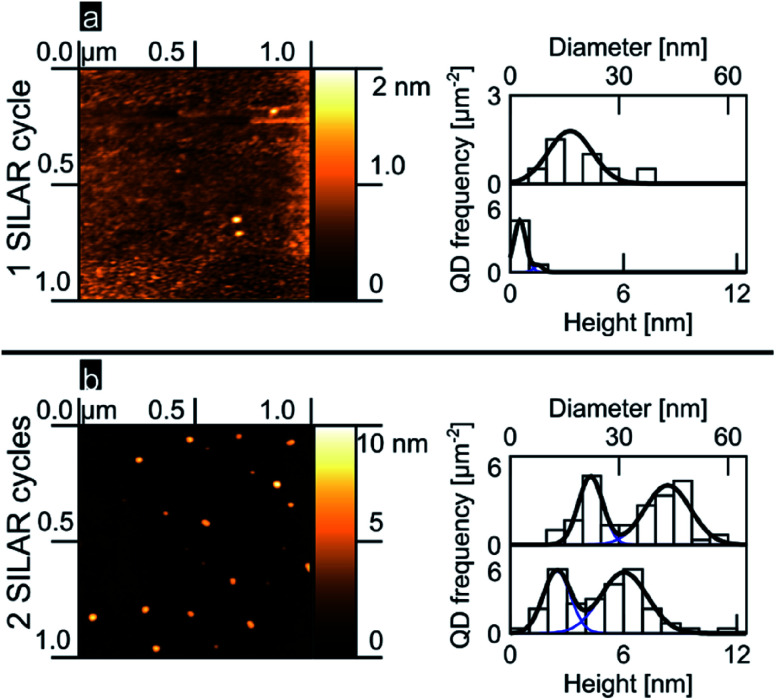
PbS SILAR on rutile (100) (a) 1 cycle, AFM image, QD height and diameter histograms and fits (b) likewise, but for 2 cycles.

We analyzed the nucleation of PbS onto the planar TiO_2_ rutile (100) surface as a function of the number of SILAR cycles. Fig. 1a and b show 1 μm^2^ AFM images accompanied by histograms of island diameter and height obtained from a total area of 3 μm^2^ (see ESI†). After 1 SILAR cycle, we find that the PbS/TiO_2_ sample is characterized by a low QD substrate surface coverage (∼3 QDs per μm^2^); within the limited sample size, the QDs reveal a narrow distribution in heights that can be described well by a monomodal distribution of QDs (see Fig. 1a). The second deposition step (2 SILAR cycles) develops clearly into a bimodal distribution of QD aspect ratios, as evident from the AFM data and the related height and diameter histograms in Fig. 1a and b. The observed transition from monomodal to bimodal QD size distributions may suggest Ostwald ripening of the quantum dots as a function of the amount of deposited material, in analogy with results commonly resolved for epitaxial growth.^5,6,8,11^ However, we note that Ostwald ripening typically leads to a reduced number of QDs, while in our work the opposite trend is observed (see Fig. 1). As such, this assignment might be premature, the emergence of a bimodal distribution after 2 cycles is likely the result of the fact that we are nucleating the QDs under saturation conditions as discussed in more detail later. Independently of the involved mechanism, to our knowledge bimodal QD size distributions have not previously been reported in mesoporous oxide matrices, despite similar precursor concentrations having been employed,^26,28,30,31,58,59^ most likely indicating that the nucleation dynamics largely depend on the available substrate surface area and/or morphology.

**Fig. 2:**
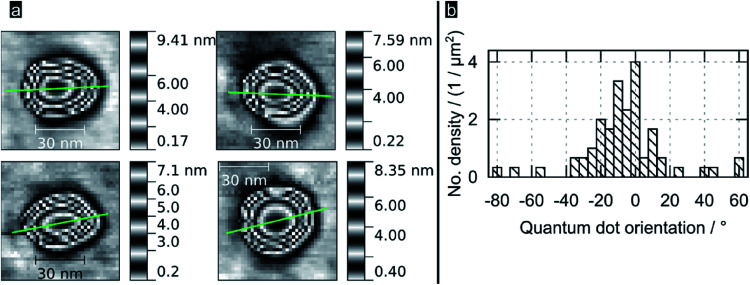
(a) Selected AFM images of PbS QDs on (100) rutile TiO_2_; green lines are the estimated orientation of their long QD axis as described in the body text (b) histogram showing the preferential orientation for the QDs nucleated by SILARs.

**Fig. 3:**
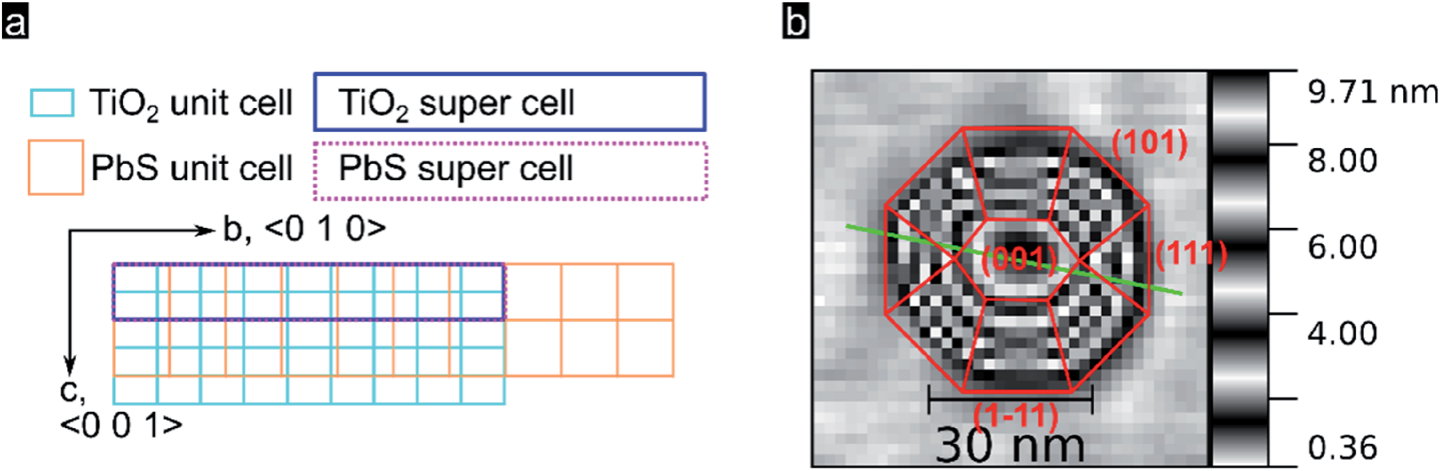
(a) Lattice of TiO_2_ rutile (100) surface, turquoise, superimposed by a lattice of PbS (100), orange. Calculated matching supercells are marked with a blue rectangle for the TiO_2_ supercell and a dotted purple rectangle for the PbS supercell. (b) Representation of a quantum dot with crystal facets inferred following Moll *et al.*^53^

Fig. 2a shows high resolution AFM images for some of the bigger QDs found in samples; the images reveal PbS QDs to be truncated pyramids with an octagonal base. The green line represents the orientation of the QD, which was obtained as the direction of the major semiaxis of an ellipse defined by the area obtained sectioning the QD at 1/4 from its total height (see ESI†). An analysis of this protocol in terms of sectioning the QDs at different heights (from 50 to 95%, see ESI†) revealed that the variance in determining the orientation per QD ranged typically between 10 and 20°, with some dots showing a variance is high as 60° (those with a section being shaped circular rather than elliptical). Fig. 2b shows a histogram of the crystal orientation (for dots sectioned at 1/4 from their total height), as indicated by the green lines in Fig. 2a and 3b. Note the preferential crystallographic orientation of the QDs peaking at about 5° *versus* the AFM scan axis. The nucleation of QDs with a preferential orientation towards the substrate indicates that the latter is acting as a seed template for QD nucleation; this is observation supports the notion of the epitaxial nature of the nucleated PbS QDs onto flat TiO_2_. The apparently large width of the obtained distribution is consistent with the inferred errors following our methodology.

The presence of a wetting layer underneath the nucleated QDs was ruled out by comparing height profiles on the plateaus of bare substrates and substrates with QDs nucleated on top (see ESI†). This was also consistent with EDX analysis that revealed an absence of PbS beyond that contained in the nucleated islands (see ESI†). The lack of a wetting layer underneath the QDs is indicative of VW epitaxial growth mode,^5^ which is consistent with the large lattice mismatch between PbS and TiO_2_ (29% between PbS and the *b*-axis of rutile (100) respectively 101% for the *a*-axis, from the lattice constants d*A* and d*B* as *ε* = (d*A* − d*B*)/d*B*.^4,5^). The observation of crystalline truncated pyramids on the planar TiO_2_ surface matches the observations made by us and others using high-resolution transmission electron microscopy for lead salt QDs sensitizing mesoporous oxide matrices;^26,37,53,62,63^ these findings demonstrate that the nature of the QD SILAR nucleation at room temperature onto an insulating titania oxide substrate is epitaxial.

In order to better understand the preferential QD crystal orientation relative to the oxide surface, we calculated the lattice mismatch for optimized superlattices derived from PbS (100), (110), and (111) low index facets and superlattices derived from the TiO_2_ rutile (100) facet following the method presented by Zur and McGill.^64^ This algorithm is conveniently applicable through the MPInterfaces Python tool set^65^ and has been used by others to elucidate epitaxial growth conditions.^22^ Details about the software stack and derived results used can be found in the (see Experimental section). As shown in Fig. 3a, for a TiO_2_ (100) supercell area of up to 400 Å^2^ we find an almost perfect lattice matching with the (100) lattice of PbS for a supercell area of 245 Å^2^ containing 7 unit cells of PbS (100) and 18 unit cells of TiO_2_ rutile (100) (vector mismatches for this optimized solution are *ε*_*u*_ = 0.3% and *ε*_*v*_ = 0.5%). These estimates of the mismatch and orientation strongly support the suitability of epitaxial growth of PbS (100) onto TiO_2_ rutile (100). Note that epitaxial nucleation was resolved for PbSe QDs (002) on Fe_2_O_3_ (003),^22^ a system characterized by larger lattice mismatches along preferential oxide axis (0.7% and 3.2% in *a* and *b* directions; 220 Å^2^ cell area). In our systems a smaller mismatch along the *c*-axis can be linked with the strain along the *c*-axis being smaller, thus leading to a preferred growth along that crystal axis. In Fig. 3b we show in red the most likely QD crystal orientation taking into account the available experimental data and modelling.

Finally, it is worth noting that under our experimental conditions, we also identified PbS rod-like structures together with the QDs shown in Fig. 1 and 2; we speculate this is due to the high precursor concentrations (PCs) employed in our relatively small-area samples.^50^ To date, region-selective growth of nanostructures by SILAR remains a challenge,.^66^ However, few reports have indeed resolved the nucleation of rod-wire like structures by SILAR, in an isolated fashion^67^ or together with QDs onto a mesoporous titania electrode.^68^ Notably, in all these reports, anisotropic growth has been linked with nucleation under saturation conditions (in agreement with our findings); specifically, some control over the nucleation of rod-wire structures was achieved by depositing the samples under non-stoichiometric conditions (with cation/anion ratios larger than 1). We speculate that the titania substrate might be catalyzing this effect, *i.e.* SILAR rinsing steps might be unable to completely remove the excess of precursors and this imbalance might promote the formation of rod-like structures. Certainly, further studies are needed to ascertain, among other aspects, the generation of a saturated bi-modal distribution of QD sizes after only two SILAR cycles. In any case, while the nucleation of our QDs is happening under saturation conditions, we believe that the main result of our work is not compromised, *i.e.* the demonstration that epitaxy of QDs onto a rutile titania surface by SILAR is feasible at room temperature. Our findings agree with work from Lau *et al.*^69^ who reported epitaxial growth of PbS nanowires using CVD on TiO_2_ rutile (001) obeying VW growth. These findings seem to illustrate that the formation of epitaxial rods is accessible by SILAR and can offer a platform for *in situ* preparation of rod-sensitized mesoporous oxides to complement *ex situ* rod sensitization schemes previously reported by others.^70^ Clearly, further work is needed to interrogate in detail the impact of immersion times, precursor concentration, substrate area to PC ratio and PC temperature on the nucleation of QDs grown by SILAR, thus paving the road for SILAR as fast, simple and reliable synthesis method for epitaxial QD growth as an addition to the established ultra-high vacuum methods. Future work should also consider other crystal facet combinations as QDs are commonly grown on mesoporous substrates that naturally exhibit more than the (100) surface of TiO_2_ rutile.^49^

## Experimental

### SILAR procedure

Chromatography grade methanol (CH_3_OH) was obtained from VWR. Analytical reagent grade acetone was obtained from Fisher Chemical. Lead nitrate (Pb(NO_3_)_2_) 99%, anhydrous sodium sulfide (Na_2_S) 98% and puriss. p.a. 2-propanol were obtained from Sigma-Aldrich. Dissolved gaseous oxygen was removed from methanol by bubbling argon through it for one hour and precursor solutions were prepared. 20 mmol L^−1^ lead nitrate and sodium sulfide solutions were used as precursor solutions. Rutile (TiO_2_) single crystals 10 mm by 5 mm by 0.5 mm, (100) faced were obtained from Crystal GmbH, Berlin, Germany. Sample substrates were sonicated in acetone for 15 minutes and for another 15 minutes in 2-propanol. SILAR was carried out in a nitrogen glovebox. Prior to starting with the SILAR cycles every substrate was immersed 30 s in fresh methanol. 1 SILAR cycle consisted of 20 s immersion in lead nitrate solution, 30 s immersion in methanol, 20 s immersion in sodium sulfide solution and 30 s immersion in methanol. After completing the desired number of SILAR cycles the substrates were immersed for 50 s in fresh methanol and dried in the glovebox. AFM images were recorded with a Bruker MultiMode equipped with a NanoScope 3a controller or a NanoScope 5 controller.

### Atomic force microscopy

The AFMs were operated in tapping mode with cantilevers of type OMCL-AC160TS (resonance frequency = (300 ± 100) kHz, typical tip radius = 7 nm) from Olympus, limiting the lateral resolution. Our data analysis is based on binning lateral data into 5 nm wide bins and height data into 1 nm wide bins. Thus the assumed accuracies are 5 nm for lateral measurements and 1 nm for vertical measurements. AFM images were analyzed using the software package Gwyddion. QDs were marked using a height threshold mask in Gwyddion, the diameter and height of the QDs were exported to generate histograms. Fitting two Gaussians to the histograms gave the diameter and height distributions included in the subpanels of Fig. 1. For details on the fitting procedure and AFM data processing see ESI.†

### Computation of lattice matched super cells

The reduction algorithm published by Zur and McGill was implemented using Python 3.5.2 and NumPy 1.11.1. This self-written implementation was used to check the correct reduction of the results from the supercell search performed with the MPInterfaces 1.2.0 package^65^ running Python 3.5.2.^71^ Further dependencies were imported in the following versions: pymatgen 3.7.1,^72^ Atomic Simulation Environment (ASE) 3.11.0,^67^ NumPy 1.11.1.^73^ To get the unit cells Structure.from_file() from the pymatgen.core.structure module was used to import cif-files obtained from the American Mineralogist Crystal Structure Database^74^ (PbS:^75^ Database Code 0011372, TiO_2_ rutile:^76^ Database Code 0009404). The Interface method from the mpinterfaces.interface module was used to calculate the lattice vectors of the two material facets investigated that are defined by the given unit cells and miller indices. Finally the best matching supercells were searched with the get_matching_lattices method from the mpinterfaces.transformations module using following constraints: max_area = 400, max_mismatch = 0.01, max_angle_diff = 1, r1r2_tol = 0.2. The *hkl* triple for TiO_2_ rutile as substrate was always set to (1, 0, 0). Three different *hkl* triples for PbS were investigated and the following lattice matching supercells were identified to fulfill the constraints. For PbS (100): supercell area *A* = 245 Å^2^, 18 substrate unit cells, 7 PbS (100) unit cells, lattice vector mismatch *u ε*_*u*_ = 0.00311, lattice vector mismatch *v ε*_*v*_ = 0.00499, angle between *u* and *v*: both 90°. For PbS (110): *A* = 299 Å^2^, 22 substrate unit cells, 6 PbS (110) unit cells, *ε*_*u*_ = 0.00311, *ε*_*v*_ = −0.00326, angle between *u* and *v*: both 90°. For PbS (111) no match was found within the used constraints.

## Conclusions

In conclusion, we have demonstrated that the nature of PbS QDs nucleated on TiO_2_ (100) by SILAR obeys epitaxy. The physics underlying the nucleation of nanocrystals at room temperature achieved by SILAR share the key characteristics of those reported for ultra-high vacuum and high-temperature methods like MBE and CVD. Our results have important implications for the design of nanostructured solar energy conversion schemes (*e.g.* solar cells and fuels); the strain accumulation during QD nucleation needs to be considered as a factor affecting structural (*e.g.* QD size and shape) and optoelectronic properties (*e.g.* bandgap) in QD sensitized oxide geometries. Owing to its rather simple preparative conditions and versatility, SILAR has great further promise for the growth of high-quality epitaxial nanostructures in liquid phase, under ambient conditions and at room temperature.

## Conflicts of interest

There are no conflicts to declare.

## Supplementary Material

NA-002-C9NA00601J-s001
