# Supplementary material for: Room-temperature solution-phase epitaxial nucleation of PbS quantum dots on rutile TiO2 (100)
Source: Nanoscale Adv. 2019 Dec 3;2(1):377–83. doi: 10.1039/c9na00601j (PMC9417650; doi:10.1039/c9na00601j)
Supplement: NA-002-C9NA00601J-s001 [file NA-002-C9NA00601J-s001.pdf]

Electronic Supplementary Material (ESI) for Nanoscale Advances.
This journal is © The Royal Society of Chemistry 2019

Supplementary Information

Room-Temperature Solution-Phase Epitaxial Nucleation of PbS Quantum Dots on Rutile TiO_2 (100)

*Stefan Kraus,[a] Mischa Bonn [a] and Enrique Cánovas *[ab]*

a. Max Planck Institute for Polymer Research, Ackermannweg 10, 55128 Mainz, Germany

b. Instituto Madrileño de Estudios Avanzados en Nanociencia (IMDEA Nanociencia), Faraday 9, 28049 Madrid, Spain

*E-mail: enrique.canovas@imdea.org

Document Structure

Reproducibility 2
AFM: Data Processing, Histogram and Fit Details 3
AFM: A Flat Substrate..5
Height Profiles of Rutile Substrates 6
AFM: Images Used for Histograms of Rutile Substrates, 1 cycle SILAR, 20 mmol L^{-1}..7
AFM: Images Used for Histograms of Rutile Substrates, 2 cycles SILAR, 20 mmol L^{-1} 8
Calculation of Quantum Dot Orientation 9
AFM: PbS Rods on Rutile..11
Elemental Composition of QDs on rutile 12

Reproducibility

During our experiments we noted that a consistent surface quality is necessary for reproducible experiments. So it is best practice to only compare samples that underwent the same surface treatment and cleaning procedures. Therefore the samples compared should be from the same cleaning batch. Furthermore, it is important to note that not all the as-received TiO_2 rutile (100) samples were as ready for epitaxy as wafer grade Si. Due to coarser mechanical polishing scratches may remain in the surface and leftover of polishing agents and other organic adsorbates can be identified on uncleaned TiO_2 rutile crystals. The sample presented and discussed here is outstanding in its surface quality, compare Figure S1a to another sample shown in Figure S1b.

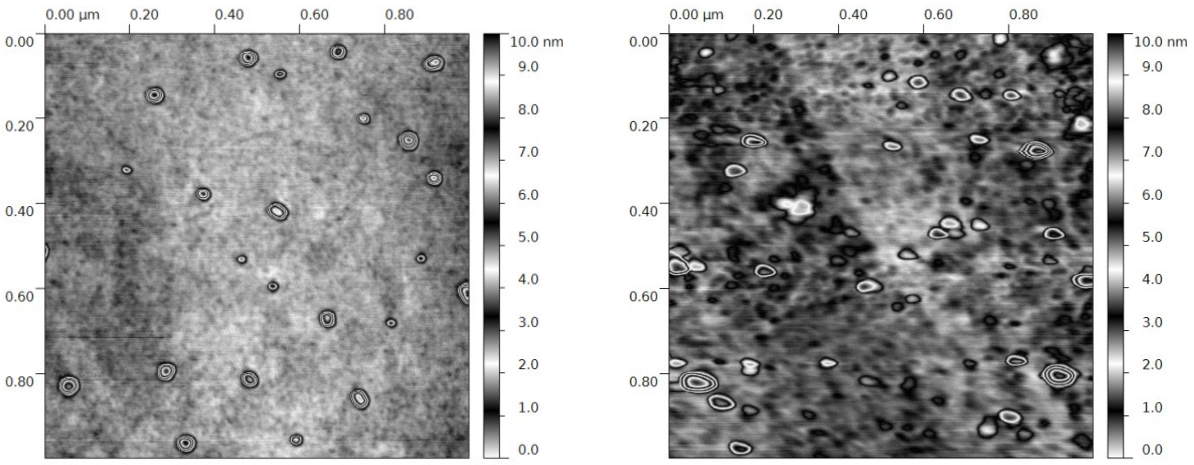

(a) (b)

Figure S1: AFM images 2 SILAR cycles, $c = 20$ mmol L^{-1} (a) sample with very flat surface showing high surface quality, (b) additional sample with coarse surface.

AFM: Data Processing, Histogram and Fit Details

1) Flatten
   a) Level data by mean plane subtraction
   b) Correct lines by matching height median
   c) Remove polynomial background with a degree up to five (usually two) in vertical an horizontal direction
   d) Shift minimum data value to zero
2) Histogram data export from Gwyddion.
   a) Mark grains by threshold and tweak mask by hand (separate grains that are two grains, but are masked as one grain).
   b) Remove grains with r_eq smaller 3 nm(1 µm with 512 dots resolution: 1 pixel = 1.9 nm) and bigger than r_eq > 30 nm.
   c) Save statistics and distributions as .txt files
3) Bin data using gnuplot. Bin width for diameter 5 nm bin width for height 1 nm.
4) Fit data with two Gaussians:

```
gaussone(x)=(area1/(fwhm1*sqrt(pi/2)))*exp(-2*((x-xmax1)/fwhm1)**2)
gausstwo(x)=(area2/(fwhm2*sqrt(pi/2)))*exp(-2*((x-xmax2)/fwhm2)**2)
doublegauss(x)=gaussone(x)+gausstwo(x)
```

Fit results are tabulated in Table S1 for QD diameter and Table S2 for QD height.

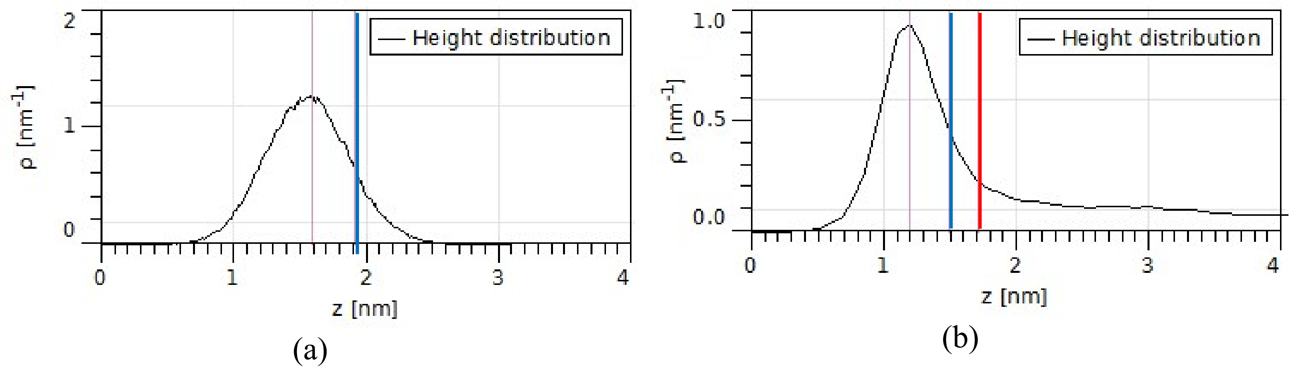

Figure S2: Height distribution black graph, FWHM blue line, height threshold for grain mask red line (a): clean Si (100), (b): 2 cycle 2 mmol L^{-1}, PbS on Si

The height thresholds are found in the following way: A clean substrate has a Gaussian like height distribution, see Figure S2a. Elements on top of this surface like the quantum dots add a tail to this Gaussian as can be seen in Figure S2b. The threshold value is chosen to be at the point where the Gaussian intersects with the residual tail, see red line in Figure S2b.

Sample	peak1 / nm	fwhm1 / nm	area1 / (nm/µm²)	peak2 / nm	fwhm2 / nm	area2 / (nm/µm²)	% area2 of graph
rutile 1 cycle, 20 mmol/L	16.5	12.0	27.0	38.1	2.5	1.7	6.1
rutile 2 cycles, 20 mmol/L	22.1	6.7	38.8	43.4	12.5	63.5	62.1

Table S1: Fit parameters for QD diameter

Sample	peak1 / nm	fwhm1 / nm	area1 / (nm/µm²)	peak2 / nm	fwhm2 / nm	area2 / (nm/µm²)	% area2 of graph
rutile 1 cycle, 20 mmol/L	0.5	0.6	2.8	1.5	0.5	0.3	8.7
rutile 2 cycles, 20 mmol/L	2.5	1.4	7.7	6.0	2.5	12.9	62.7

Table S2: Fit parameters for QD height

AFM: A Flat Substrate

After the cleaning process outlined in the main text the samples show terraces on their surfaces as shown, for example, in Figure S3. This clean state is referred to as 0 cycles SILAR.

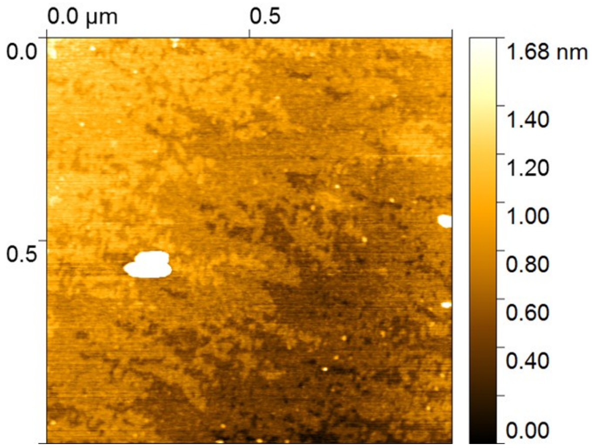

Figure S3: Flat surface of a cleaned sample before the SILAR process, 0 cycles

Height Profiles of Rutile Substrates

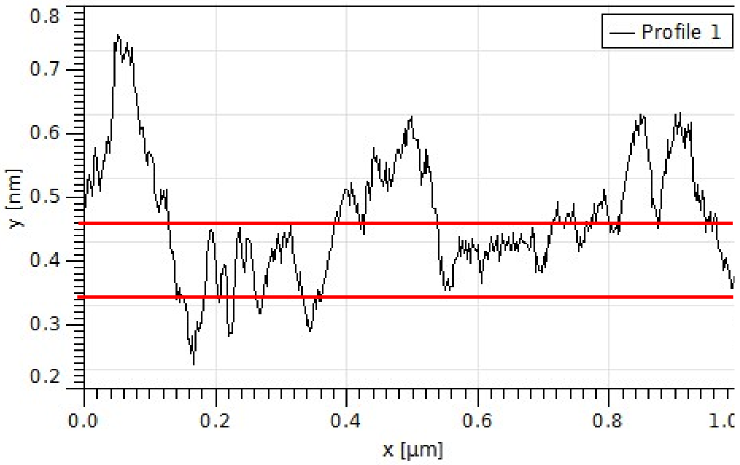

(a)

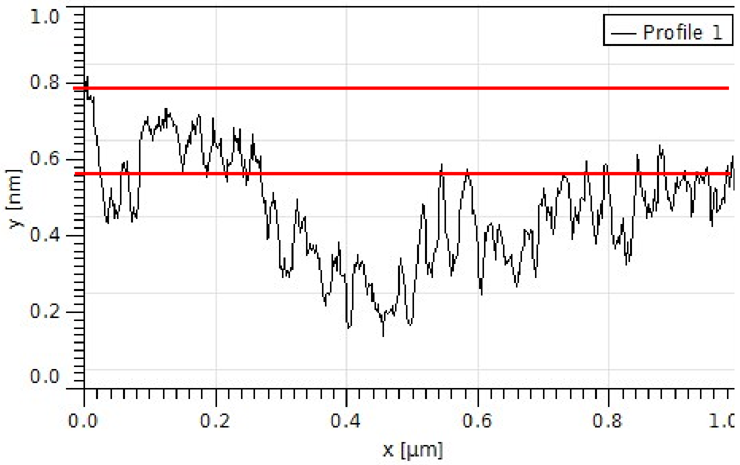

(b)

Figure S4: Line profile of rutile substrate without grains (a): 0 cycles, (b): 2 cycles shown in Figure S6

The spacing of the red lines in Figure S4a and Figure S4b is 0.12 nm. As the random height changes are still present after the SILAR treatment and expected terraces are missing, Stranski-Krastanow growth can be ruled out for depositing PbS on rutile using SILAR.

AFM: Images Used for Histograms of Rutile Substrates, 1 cycle SILAR, 20 mmol L^{-1}

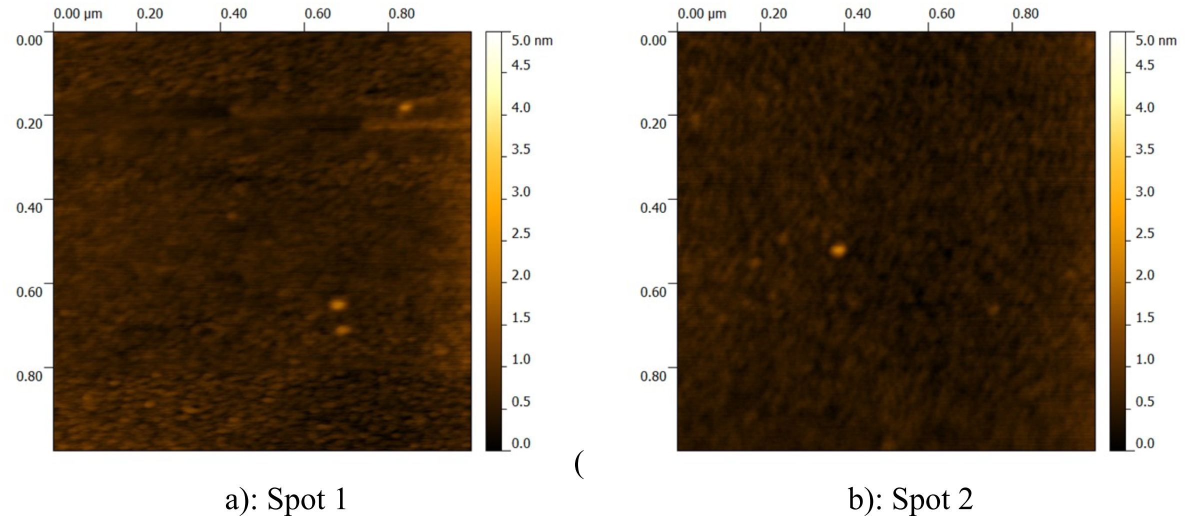

((

a): Spot 1 b): Spot 2

Figure S5: AFM images of PbS SILAR on rutile used for histograms, 1 cycle, c = 20 mmol L^{-1}.

AFM: Images Used for Histograms of Rutile Substrates, 2 cycles SILAR, 20 mmol L^{-1}

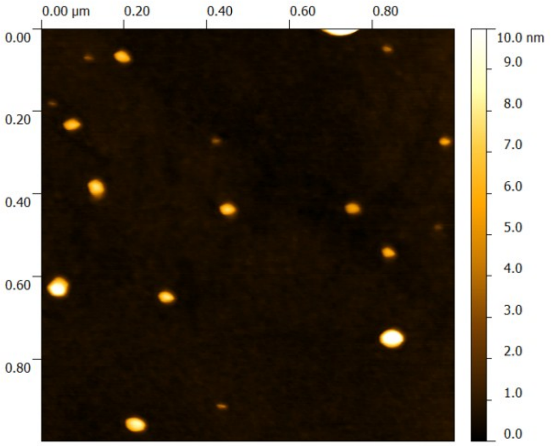

(a): Spot 1

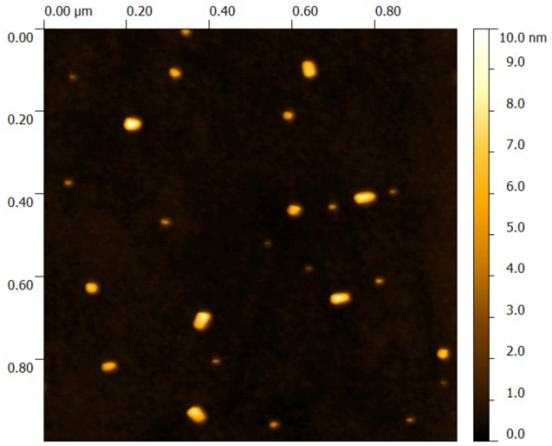

(b)

: Spot 2

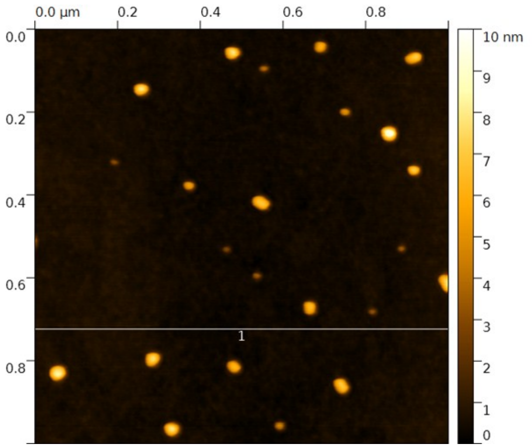

(c): Spot 3

Figure S6: AFM images of PbS SILAR on rutile used for histograms, 2 cycles, c = 20 mmol L^{-1}.

Calculation of Quantum Dot Orientation

For this work the orientation of a quantum dot was defined as the direction of the major semiaxis (φ_{e1}) of an ellipse that has the same second order moments in the horizontal plane as the quantum

dot. For calculation of the QD orientation the Python 2.7.10 console of Gwyddion 2.41, often referred to as pygwy, was used. Python code, performing the following tasks for all individual QDs in an image, was run after the QDs were marked as described in section AFM: Data Processing, Histogram and Fit Details step 2) Histogram data export from Gwyddion.

1) Select the top 25 % of the QD to avoid influence from the heavily convoluted QD base.
2) Get φ_{e1} for the selected area (as stored in an object of class DataField as provided by the Gwyddion delivered Python module `gwy`) using the instance method `grains_get_values` for the quantity `GRAIN_VALUE_EQUIV_ELLIPSE_ANGLE`.
3) Export QD orientation φ_{e1} together with other QD properties, e.g. equivalent disc radius, absolute height minimum and maximum, as text file in comma separated value format.

<u>Error estimate on QD orientation:</u>

We evaluated our methodology with the aim of estimating an error in the inferred orientations. For doing so, we have followed the same protocol but calculating orientations of the major semiaxis of the ellipse at different QD heights ranging between 50 and 95% (figure S7 left). As shown in the figure for a single QD, selecting different heights does have a marked influence on the obtained orientation following our methodology. In the right panel of figure S7 we present a summary of this analysis taken over 15 QDs. Taking into account the error estimates, all analyzed QDs do have the same orientation, ~8 degrees versus the AFM scan axis with an associated variance ranging between 10 and 60 degrees. We found that those dots strongly deviating from the main group had the largest error bars, this was a consequence of a poorly define elliptical shape for the plane cut of QD at different heights; the section provides for these dots a circular, rather than elliptical, shape.

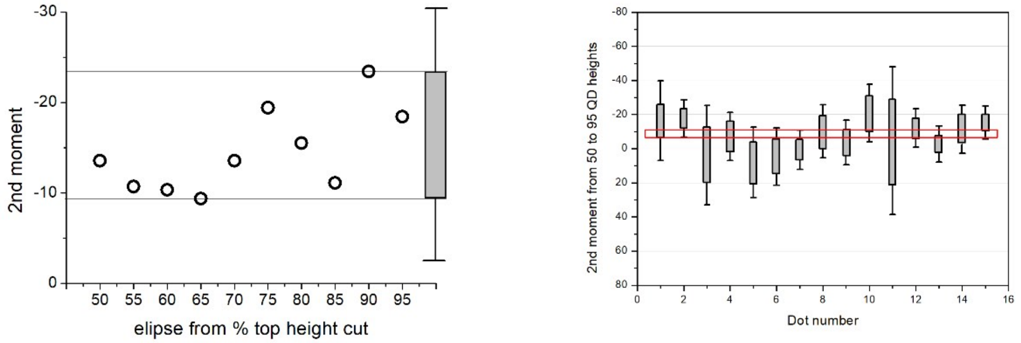

Figure S7: (left) orientations of the major semiaxis of the ellipse that has the same second order moments in the horizontal plane as the quantum dot at different heights ranging between 50 and 95%. (right) idem for 15 QDs, red line represents most probable orientation of the dots vs AFM scan axis.

While we cannot rule out that some physicochemical factors as titania surface contamination, the presence of terraces or scratches induced by mechanical polishing of the substrates might have affected our results, we primarily assign the apparently large dispersion found in QD orientation to the limitations of the employed mathematical methodology. The resolution of the images and factors affecting the AFM images as drifts and/or sharpness of the tips are likely responsible for the apparently large dispersion of orientations.

AFM: PbS Rods on Rutile

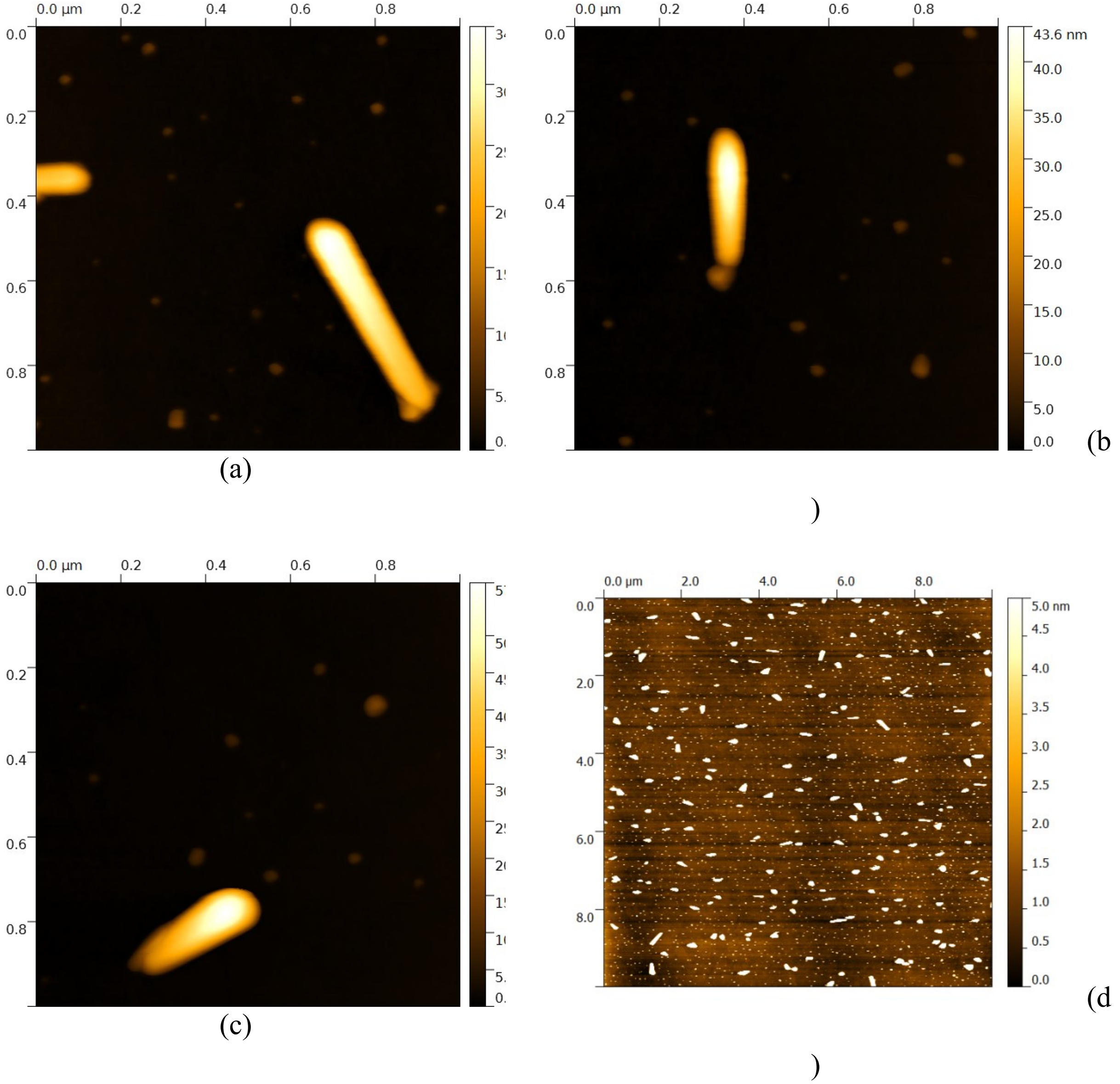

Figure S8: AFM images of PbS on TiO_2, with rods (a) Spot 1, (b) Spot 2, (c) Spot3, (d) Overview

Elemental Composition of QDs on rutile

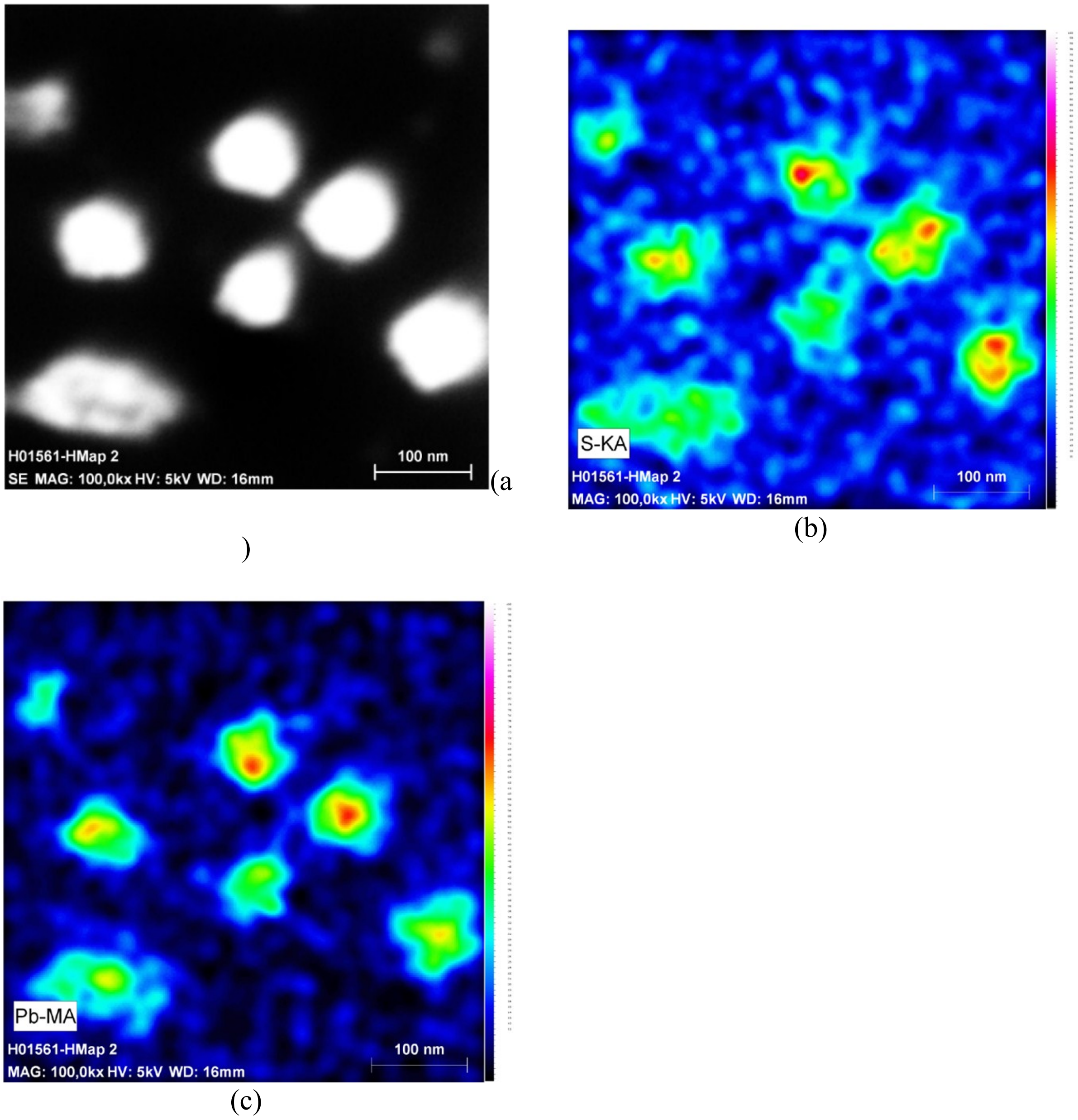

Figure S9: Elemental Composition of QDs on rutile (a) SEM image, (b) EDX sulfur hyper map (c) EDX lead hyper map
